# Comparing the Effects of Lovastatin and Cornus Mas Fruit on Fibrinogen Level in Hypercholesterolemic Rabbits

**Published:** 2010

**Authors:** Sedigheh Asgary, Mahmoud Rafieian-Kopaei, Azadeh Adelnia, Somayeh Kazemi, Fatemeh Shamsi

**Affiliations:** 1 Associate Professor of Pharmacognosy, Isfahan Cardiovascular Research Center, Applied Physiology Research Center, Isfahan University of Medical Sciences, Isfahan, Iran; 2 Professor of Pharmacology, Shahrekord Medicinal Plants Research Center, Shahrekord University of Medical Sciences, Sharekord, Iran; 3 MSc, Animal Physiology, Department of Biology, University of Payam-Noor. Isfahan, Iran; 4 MSc, Animal Physiology, Department of Biology, The University of Isfahan, Isfahan, Iran

**Keywords:** Atherosclerosis, Fibrinogen, Lovastatin, Rabbits

## Abstract

**BACKGROUND:**

Atherosclerosis, which is a result of gradual deposition of lipids in the lower part of blood vessel endothelium, is the leading cause of mortality and morbidity around the world. It has been proved that some inflammatory blood markers such as fibrinogen can predict the risk for cardiovascular disease conditions, not only in cardiovascular patients, but also in those who do not have any manifestations of the atherosclerotic development. In this study, the effect of cornus mas l. was evaluated on fibrinogen of hypercholesterolemic rabbits and it was also compared with lovastatin drug.

**METHODS:**

In this study, 25 New Zealand adult male rabbits were randomly divided into five groups of five. They were treated for 60 days by 5 different diets, namely basic, high cholesterol, regular plus 1 g/kgBW cornus mas L. powder, high cholesterol plus 1 g/kgBW cornus mas L. powder, and high cholesterol plus 10 mg/kgBW lovastatin. At the beginning and at the end of this period, blood samples were collected from the rabbits and their serum fibrinogen levels were measured.

**RESULTS:**

Cornus mas L. powder and lovastatin significantly decreased fibrinogen levels in comparison with high cholesterol group (P < 0.05). Furthermore cornus mas L. powder could reduce the fibrinogen level more than lovastatin (P < 0.05).

**CONCLUSION:**

The results indicated that consumption of cornus mas L. might be beneficial in atherosclerotic patients due to its reducing effects on fibrinogen.

## Introduction

Atherosclerosis and its side effects are among the major mortality causes in the world.^1^ Research shows that homeostatic systems play an important role in pathogenesis of atherosclerosis vascular disease.^2^, ^3^

Fibrinogen is linked with cardiovascular diseases via the two processes of inflammation and thrombosis, which are the two major processes in the progress of atherosclerosis.^4^

Similar to C-reactive protein (CRP), fibrinogen is an acute phase reactor, which is more likely to be an inflammatory symptom rather than having a role in pathogenesis of coronary events. Fibrinogen synthesis in liver can increase up to four times during an inflammatory reaction or infection. In addition to inflammation, a number of other factors are also known as fibrinogen level regulators. Fibrinogen levels are higher in diabetes and hypertension patients, and also among obese people, or those with low physical activity lifestyles.^5^ Moreover, fibrinogen levels increase as low density lipoprotein (LDL) or a lipoprotein (LP (a)) levels rise; its level is also higher in smokers.^6^

Along with coagulation, fibrinogen has other different functions through which it can act as a cause for cardiovascular diseases, including atherosclerosis. These functions are as follow:

1. Regulation of cell (including vascular smooth muscle cells) adherence, chemotaxis, and reproduction
2. Arterial contraction in atherosclerotic injury sites
3. Determining blood fluidity and density (blood adherence)^5^, ^7^

Although contrasting reports exist about fibrinogen changes in statin therapy, some researches suggest that lipid reduction medications, such as statins, can reduce the harmful cholesterol level in the body, but have a little effect on the reduction of fibrinogen level, as a cardiovascular disease risk factor.^8^ Furthermore, the side effects of these medications^9^, ^10^ are other reasons to search for effective herbal compounds. Cornelian cherry, scientifically named cornus mas L., grows in different parts of Europe and Asia, including Iran. The fruit is full of glucose, fructose, lactose, organic acids and tannin. It also contains different kinds of antioxidant compounds, including flavonoids, antosianins, anthocyanin, and many vitamins.^11^–^13^

Having the qualities of cornus mas L. in mind and knowing that fibrinogen is considered as a risk factor for atherosclerosis development, and also based on previous studies about anti-inflammatory and antithrombotic effects of flavonoid compounds, we have decided to examine the effectiveness of cornus mas L. fruit on hyperfibrinogenemia in hypercholesterolemic rabbits with a high cholesterol diet.

## Materials and Methods

Cornus mas L. was purchased at the fruit market, and the quality and species of it were determined by a botanist in a herbarium in Shahrekord's medicinal plants center. After the fruits were washed and their seeds were removed, they were dried for 1 month and finally used as a powder.

### Measurement of the effective components in phenolic extracts

Total phenol content was determined using Folin-Ciocalteu reagent method in terms of gallic acid. Total flavonoid and flavonol contents were calculated in terms of rutin, using a method suggested by Pourmorad et al (2006) and a method suggested by Loziene et al,^14^, ^15^ respectively.

### Categorizing and treating the rabbits

Twenty five adult male New Zealand rabbits, weighing 2-2.5 kg, were purchased from Razi institution in Karaj and were transported to Shahrekord medicine school. The rabbits were held in standard light and temperature and were treated with basic diet for 2 weeks in order to adapt them to the new environment.

The rabbits were then randomly categorized into 5 groups of 5. They were treated as follows for 60 days: the first group with a basic diet, the second with a high cholesterol (1%) diet, the third with a basic diet containing cornus mas L. powder (1 g/kgBW daily) diet, the forth with a high cholesterol (1%) containing cornus mas L. powder (1 g/kgBW daily) diet, and the fifth with a high cholesterol containing lovastatin (10 mg/kgBW daily) diet.

During this 60-day period, the rabbits could freely access the food and water. Cornus mas L. powder (1 g/kgBW) was fed to rabbits using gavage, along with 5 cc water.

In high cholesterol diets, cholesterol made by Merc factory in Germany was gavage fed to the rabbits.

### Measurement of biochemical factors

Before starting the diets and after the period of the study, the rabbits were fasting for 12 hours. Then, a blood sample was taken and the plasma was used to examine the fibrinogen factor.

Fibrinogen was measured by means of a Mahsayaran kit, using coagulative method.

### Statistical analysis

The results have been statistically analyzed in the form of mean ± SD. In order to examine the biochemical results and to compare the mean values for different experimental groups, ANOVA test and SPSS_15_ software were used. P < 0.05 was considered as significant. The graph was drawn by Excel software.

## Results

Each 100 g of cornus mas L. powder yielded 11 ± 0.4 g ethanolic extract. The calculated levels for flavonoid, flavonol, and phenol were 17.98 ± 1.9 mg/g, 11.13 ± 5.80 mg/g, and 215.56 ± 2.88 mg/g, respectively.

**Figure 1:**
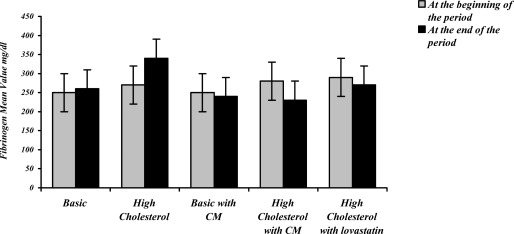
The effects of cornus mas L. (CM) fruit and lovastatin on serum fibrinogen level in hypercholesterolic rabbits (the data is presented as mean ± SD, the study type was experimental) *The significant difference between the mean value of fibrinogen in high cholesterol and basic groups (P < 0.05) **The significant difference between the mean value of fibrinogen in high cholesterol+interventional treatment and high cholesterol groups ***The significant difference between the mean value of fibrinogen at the beginning and at the end of the period of study in each group

The mean values for biochemical factors among the five groups did not have any significant difference at the beginning of the period (Figure 1). As the graph shows, the high cholesterol diet resulted in a significant increase in the density of fibrinogen, compared to the basic diet (P < 0.05). Moreover, treating the rabbits having a high cholesterol diet with cornus mas L. powder or lovastatin caused a significant reduction in fibrinogen, as compared with the basic high cholesterol diet (P < 0.05). The fibrinogen level in the group receiving the basic diet containing cornus mas L. had an insignificant reduction compared to the group receiving the basic diet. An insignificant reduction in fibrinogen level was also observed when the high cholesterol diet containing cornus mas L. was compared with the high cholesterol diet containing lovastatin.

## Discussion

The results obtained from this study indicate that in high cholesterol diets, cornus mas L. and lovastatin can reduce the fibrinogen level.

Fibrinogen is an inflammatory and coagulation risk factor and has an important role in the complexity of atherosclerosis process.^16^ The enforcement of platelet aggregation and formation of fibrin coagulum are among the primary functions of fibrinogen during homeostasis process in the arterial injury site.^17^ It has been reported that fibrinogen oxidation and its fibrinolyze residuals stimulate platelet aggregation and IL-6 increment,^16^ and therefore the reducing effects of cornus mas L. on fibrinogen levels can be due to the participation of its components in inflammatory or coagulative processes.

There have been studies showing the effects of herbal diets on the processes of inflammation and fibrinolyze. These compounds are effective in the reduction of coagulative factors, or the augmentation of fibrinolyze via reducing blood coagulation by means of decreasing fibrinogen levels, increasing fibrinolyze and prothrombin time, and also preventing platelet aggregation.^18^, ^19^ The anti-inflammatory and arterial protective effects^20^ of anthocyanins, which are among the most important antioxidants in cornus mas L. fruit,^21^, ^22^ along with their usefulness in the excretion of free radicals, have been investigated.^8^, ^23^, ^24^

Research shows that the consumption of antioxidants will increase the antioxidantal capacity and decrease cardiovascular disease development chance, and also the risk associated with raised fibrinogen level.^25^ As a result, it is probably true that the antioxidant compounds in cornus mas L. fruit, including vitamins and phenolic compounds, would prevent free radical action and fibrinogen oxidation and its fibrinolyze residuals, which stimulate platelet aggregation and increase inflammatory cytokines.

Inflammation and platelet aggregation will increase fibrinogen production in the liver by 4 times. On the other hand, Fibrinogen level increases in response to interleukins 1 and 6 (cytokines produced in arterial disorders).^26^ Evidence suggests that the bare matrix under an injured endothelium contains a great amount of pre-coagulation compounds, including collagen. In the process of absorption and activation of platelets, collagen acts like a ligand. In other words, just like a strong antagonist, collagen causes the release of platelet granules and the presentation of active ligands, such as IIb- IIIa. Platelet granules contain VWF (Von Willebrand Factor) and fibrinogen which act as a connecting bridge between the collagen under endothelium and glycoprotein platelet receptor (GPTb). The process of platelet aggregation continues as fibrinogen connects a big number of platelets to each other via GPIIb-IIIa platelet receptors.^27^ Research shows that in the inflammation and arterial wall injury site, anthocyanins and anthocyanidins affect the structure and metabolism of collagen and cause stronger collagen fibers and better crosslink among them (via affecting proline hydroxylase enzyme), and actually prevent collagens from attaching to platelets in the primary stages of inflammation and arterial injury.^28^ In addition, anthocyanic extract stops inflammation, and thus atherosclerosis progress, by preventing elastase enzyme (a collagen destructive enzyme) from action.^29^ It has been observed that polyphenols existing in cocoa reduce the inflammatory cytokinines and inhibit platelet activity and epinephrine gene (a platelet aggregation agonist that is produced at platelet aggregation site) expression and also glycoprotein IIa/IIIbv on the platelet surface; in addition, they reduce P-Selection expression (as an inflammatory agent).^30^

Research shows that delphinidin and pelargonin glycosides, existing in cornus mas L. fruit, inhibit cyclooxygenase inflammatory enzymes activity^31^, ^32^ and therefore reduce the levels of pre-inflammatory compounds and cytokinines. Recent studies have demonstrated that polyphenols such as catechin and quercetin, stop NADPH oxidase enzyme (existing in platelets), thus inhibiting the production of O_2_^-^ and increasing the biological power of NO, and consequently regulate the glycoprotein pine receptors on platelet membrane surface (GPIIb-IIIa) which in turn inhibits the platelets activation and their adherence during the inflammation and thrombosis processes 33.

Consumption of lovastatin has also led to a reduction in fibrinogen level compared to the high cholesterol group.

Statins are a group of antihyperlipidemic compounds and anti-inflammatory effects are amongst their most important pleiotropic qualities. These effects are due to the ability of statins in preventing mevolonic acid formation, whose reduction results in a decrease in cholesterol level and some other isoprenoid inflammatory mediators.^34^ It was also observed that in hyperlipidemic patients, statins reduce the platelet adhesive molecules, ICAM-1 and P-Selection; they also decrease MCP-1 expression, interleukins 1 and 6 production, and oxygenase-2 cycle gene expression in human endothelial cells.^35^–^37^ In fact, statins reduce platelet clot and cardiovascular diseases via decreasing inflammatory agents and affecting homeostase process and NO production by endothelial cells.^38^, ^39^

## Conclusion

In basic and high cholesterol diets, cornus mas L. powder consumption results in fibrinogen level reduction which is an indicator of this fruit's anti-inflammatory and anticoagulative effects. The effectiveness of this fruit on the mentioned biochemical parameter equals to (or even surpasses) lovastatin with a dose of 10 mg. Considering the usefulness of cornus mas L. on reducing fibrinogen (which is an important inflammatory or coagulative agent, and also a major risk factor for cardiovascular diseases), further investigations on this subject seem necessary.
